# Fabrication of Hybrid Nanofibers from Biopolymers and Poly (Vinyl Alcohol)/Poly (ε-Caprolactone) for Wound Dressing Applications

**DOI:** 10.3390/polym13132104

**Published:** 2021-06-26

**Authors:** Sibusiso Alven, Blessing Atim Aderibigbe

**Affiliations:** Department of Chemistry, University of Fort Hare, Alice Eastern Cape, Alice 5700, South Africa; 201214199@ufh.ac.za

**Keywords:** wound dressing, biopolymers, poly (vinyl alcohol), poly (ε-caprolactone), electrospinning, nanofibers

## Abstract

The management of chronic wounds is challenging. The factors that impede wound healing include malnutrition, diseases (such as diabetes, cancer), and bacterial infection. Most of the presently utilized wound dressing materials suffer from severe limitations, including poor antibacterial and mechanical properties. Wound dressings formulated from the combination of biopolymers and synthetic polymers (i.e., poly (vinyl alcohol) or poly (ε-caprolactone) display interesting properties, including good biocompatibility, improved biodegradation, good mechanical properties and antimicrobial effects, promote tissue regeneration, etc. Formulation of these wound dressings via electrospinning technique is cost-effective, useful for uniform and continuous nanofibers with controllable pore structure, high porosity, excellent swelling capacity, good gaseous exchange, excellent cellular adhesion, and show a good capability to provide moisture and warmth environment for the accelerated wound healing process. Based on the above-mentioned outstanding properties of nanofibers and the unique properties of hybrid wound dressings prepared from poly (vinyl alcohol) and poly (ε-caprolactone), this review reports the in vitro and in vivo outcomes of the reported hybrid nanofibers.

## 1. Introduction

Skin is the largest organ of the human body and it is an essential barrier featured with immunologic, sensorial, and protective capability [1]. Due to its exposure to the external environment, the skin is the most vulnerable body organ to a diversity of external factors that can cause different skin injuries. Several negative factors delay the wound healing process, including bacterial infections, venous and arterial insufficiency, malnutrition, smoking, obesity, and poor blood circulation, and underlying conditions such as diabetes and cancer [2]. Chronic wounds affect approximately 1–2% of the European and United States (US) population [3]. These wounds result in a negative socio-economic impact. For example, over 184 million pounds was spent in England on wound dressing materials in 2012. Approximately 20 billion US dollars is spent every year in the United States of America to treat severe wounds [4]. In 2014, an annual cost of approximately USD 2.8 billion was spent worldwide on wound dressings, and this is expected to increase to USD 3.5 billion in 2021 [5]. Furthermore, the market research report in 2018 predicted that wound dressing products would reach USD 15 billion by 2022 globally. The advanced wound care market targeting chronic ulcers and surgical wounds is expected to exceed USD 22 billion by 2024 due to increasing cases of chronic wounds [5].

**Figure 1:**
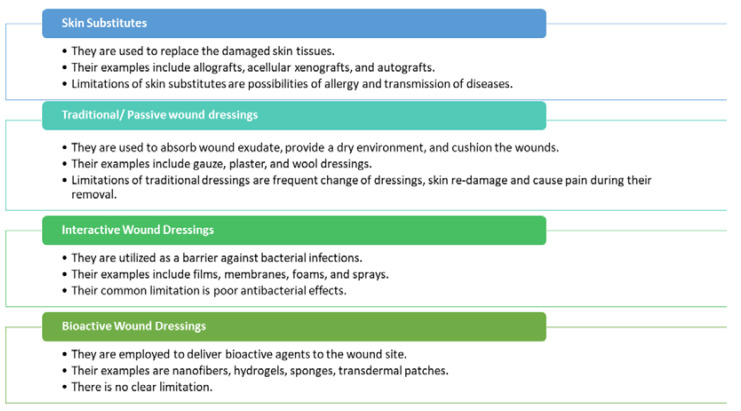
Classification of wound dressings.

Most patients suffer from poor wound healing of chronic wounds, a primary cause of mortality. There is an urgent need to develop wound dressings suitable for the accelerated wound healing mechanism of chronic wounds. These wound dressings should be able to allow gaseous exchange, effective for the absorption of excess wound exudates, non-toxic, biocompatible, prevent microbial invasion, and non-allergenic [6]. The presently used dressing materials are shown in Figure 1. Wound dressings are classified as skin substitutes, traditional dressings, interactive dressings, and bioactive dressings [4].

Skin substitutes are wound dressings used to replace the disrupted skin tissue. Examples of skin substitutes include allografts, acellular xenografts, and autografts [7]. The most skin substitute that is frequently utilized for the management of chronic wounds is allografts. The shortcomings of skin substitute dressings include allergy, possibilities of disease transmission, etc. [6]. The traditional wound dressings such as gauze, plaster, etc., absorb wound exudates, provide a dry environment and cushion for the wounds. However, their use is limited by the need for frequent changes resulting in further skin injury and pain [8]. Interactive dressings offer several advantages such as enhancing granulation and re-epithelialization, provide a moist environment for the wound, modify the physiology of the lesion environment, and improve water vapor transmission rate with excellent mechanical performance [9]. Some examples of interactive dressings are spray, films, foams, nanofibers, and sponges. Bioactive wound dressings are loaded with bioactive agents such as antibiotics, growth factors, nutrients, nanoparticles, vitamins, plant extract, and other natural biomaterials.

Bioactive wound dressings are prepared from biopolymers such as chitosan, alginate, silk pectin, elastin, collagen, fibroin, and hyaluronic acid. Examples of bioactive dressings include nanofibers, membranes, wafers, foams, hydrogels, sponges, and films [10]. Nanofibers prepared from the above-mentioned biopolymers demonstrate unique features in the wound healing applications, such as patient compliance, good biocompatibility and biodegradable, better antibacterial effects, and controlled drug delivery mechanism [11]. The main limitation of biopolymer-based nanofibers is their poor mechanical performance. An ideal wound dressing must demonstrate good mechanical properties (i.e., good elasticity, flexibility, durability, pliability, and stress resistance) for easy application and removal without causing further injury. Furthermore, the mechanical properties of wound dressing material must imitate those of the human skin for good biocompatibility.

The poor mechanical properties of biopolymer-based nanofiber wound dressings can be significantly improved by crosslinking them with synthetic polymers. The synthetic polymers that are frequently used for cross-linking include poly (lactic acid) (PLA), poly(D,L glycolic-co-lactic acid) (PLGA), poly (glycolic acid) (PGA), poly (ԑ-caprolactone) (PCL), poly(vinyl pyrrolidone) (PVP), poly(ethylene glycol) (PEG), poly(vinyl alcohol) (PVA), polyurethanes (PUs), etc. This review article will be focused on hybrid nanofibers prepared from a combination of biopolymers and synthetic polymers (poly (vinyl alcohol)/ poly (ε-caprolactone)) for wound healing application. Several studies have demonstrated that the biopolymer-based hybrid nanofibrous wound dressings possess interesting properties (e.g., imitating extracellular matrix, good antimicrobial effects, good wound healing properties, etc.) for improved wound care.

## 2. Classification of Wounds and the Phases of the Wound Healing Process

Wounds are classified based on their time of healing and depth. The wounds are classified based on their duration of healing as acute and chronic wounds [12]. Acute wounds heal between a period of about 2 to 3 months [13]. They are caused by corrosive chemicals, electrical damage, accidental injury, and surgical procedure. Improper care of acute wounds, microbial invasions, or underlying conditions such as diabetes can result in them becoming chronic wounds. Chronic wounds are injuries that fail to heal over a prolonged period [14]. The factors that can contribute to chronic wounds include diabetes, cancer, prolonged bed rest, smoking, obesity, and age [15]. Wounds can also be classified based on their depth in the skin as superficial wounds, partial-thickness wounds, and full-thickness wounds [16]. Superficial wounds disrupt the epidermal lining of the skin and its components [17,18]. The partial-thickness wounds result from damage to both the epidermal and dermal layer of the skin, while in the full-thickness wounds, there is a disruption of the layers and the deep tissues [19].

**Figure 2:**
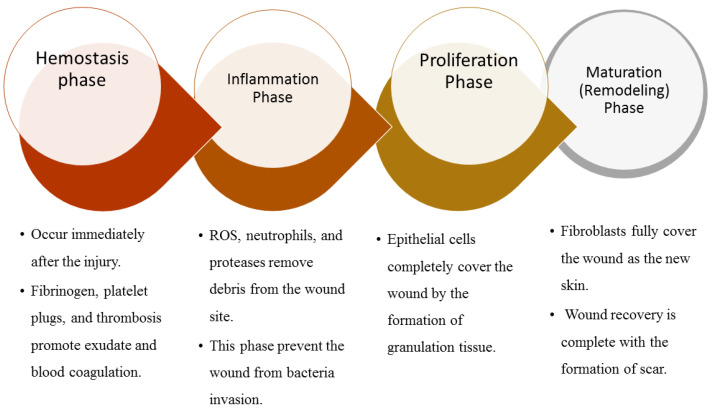
Phases of wound healing.

The wound healing process of the wounds mentioned above is a complex process that involves cellular and biochemical events. It involves four sequential and sometimes overlapping phases: hemostasis phase, inflammatory phase, proliferation phase, and remodeling phase (Figure 2) [20,21]. These wound healing phases can be delayed in some cases, especially during the inflammation phase. The hemostasis phase happens instantly after the injury. It is characterized by the presence of fibrinogen (a significant component of the connective skin tissue), thrombosis, and platelet plug caused by platelets, which stimulate the coagulation of exudate and blood to terminate bleeding [22,23]. The inflammatory phase usually occurs concurrently with the hemostasis phase [24]. It is where the reactive oxidative species (ROS), neutrophils, and proteases that are released by phagocytic cells help in the removal of debris from the wound site and the prevention of bacterial infection on the wound [25]. The exudate in this phase causes pain, swelling, and redness of the wound or even the edema or erythema of the skin [26].

The third phase of the wound healing process is the proliferation phase. The macrophages release epithelial cells, growth factors, keratinocytes, fibroblasts, and cytokines to replace the damaged tissues and epithelial cells that invade the wound site to completely cover the wound by forming granulation tissue [24,27]. The last phase is remodeling which is also called the maturation phase. In this phase of the wound healing process, fibroblasts fully cover the wound as the new skin epithelial layer, and the wound closure is complete with the formation of a scar [28]. It is essential to select suitable wound dressing for each phase of the wound healing, and the dressing must demonstrate good mechanical features. Some good features of an ideal wound dressing include good mechanical performance, excellent antimicrobial effects, good water vapor transmission, permeability for gaseous exchange, the transmission of nutrients, swelling capacity, high porosity, good cellular adhesion, and non-toxicity and excellent biocompatibility [29].

## 3. Poly (Vinyl Alcohol) and Poly (ε-Caprolactone) in Wound Healing Applications

**Figure 3:**
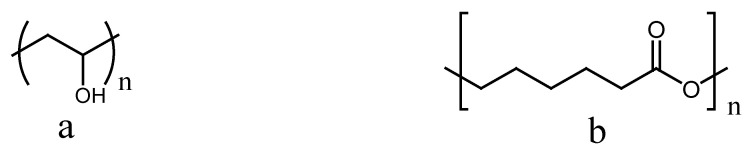
Chemical structures of synthetic polymers (**a**) poly (vinyl alcohol) and (**b**) poly (ε-caprolactone).

Poly (vinyl alcohol) (PVA) is a non-carcinogenic synthetic polymer produced from vinyl acetate by hydrolysis, alcoholysis, or aminolysis (Figure 3a). It is used in a diversity of biomedical applications due to its biocompatibility, biodegradability, non-toxicity, hydrophilicity, and low tendency for protein adhesion [30]. It has been broadly utilized for tissue regeneration and drug delivery applications. Besides its excellent hydrophilic nature and fluid absorption ability, it also demonstrates an outstanding capability to be manipulated in the form of fibers, particles, sponges, textiles, and films [27]. It has been employed in the formulation of polymer-based wound dressings to treat chronic wounds and acute injuries. Due to its attractive properties, PVA has been frequently utilized in biopolymer-based dressings to improve the mechanical performance of the dressing for wound healing and skin regeneration. Due to the strong affinity of PVA wound dressings for binding with glucose, some researchers suggested that it could be utilized to develop wound dressings to treat diabetic wounds [31]. However, plain PVA–based wound dressings have an incomplete hydrophilic feature with insufficient elasticity and rigid structure, which limit its application alone as wound dressing scaffolds [32]. Furthermore, some PVA–based wound dressings suffer from poor stability in water [33]. Among the numerous wound dressing materials reported, wound dressings formulated from PVA combined with some biopolymers and some other synthetic polymers display attractive features, such as excellent biocompatibility, biodegradability, sustained drug release profiles, etc. [34].

Polycaprolactone (PCL) is a synthetic polymer that belongs to the class of aliphatic polyesters (Figure 3b) together with polylactide (PLA), polyglycolic acid (PGA), and poly(lactic-co-glycolic acid) (PLGA) [27]. PCL is a biocompatible and biodegradable polymer that has been investigated for wound healing and tissue regeneration applications. It stimulates quicker wound healing and decreases inflammatory infiltration [27]. Nonetheless, PCL biodegrades at a significantly slower rate in comparison with PLGA, PGA, and PLA. This slow biodegradation causes PCL to be less attractive for this type of biomedical application but more attractive for controlled-release, sutures, and long-term implant applications [35]. Therefore, the combination of biopolymers with PVA or PCL to prepare hybrid nanofibers via electrospinning technique can result in interesting excellent properties that would be very suitable for wound healing and skin regeneration applications. The main limitation of PCL is poor cell adhesion and growth resulting from its hydrophobic surface [36]. Therefore, it essential to blend PCL with natural polymers to enhance its cellular attachment and proliferation. The other disadvantage of PCL is weak antimicrobial effects that can be overcome by loading antibacterial agents such as antibiotics and metallic nanoparticles [37].

## 4. Electrospinning Technique and Properties of Nanofibers

Although there are many techniques employed for the production of nanofibers and nanofibrous materials, the electrospinning method is the most employed, simpler, cost-effective, more environmentally friendly method [38,39]. Encapsulation of different types of bioactive agents can be loaded into nanofibers and nanofibrous materials for the controlled release of drugs in biomedical applications. Most of the electrospun nanofibrous scaffolds are non-toxic, and the apparatus is simple and made of three components (voltage system, spinneret system, and collecting system) that make it cost-effective. Electrospinning uses high voltage electric fields to produce nanofibers with diameters in several nanometers and micrometers [40]. The electrospinning parameter can be manipulated based on the required structures and morphologies of nanofibers [41]. The spinneret designs promote the generation of the near-field, and the coaxial electrospinning process has been developed to deposit nanofibers in a continuous, controllable, and direct manner [42]. This method can produce nanofibers with different structures such as hollow, three-dimensional, core-shell, and nanowire-in-microtube fiber materials formed by modifying the configuration of the spinning apparatus [43].

Electrospinning technique with high versatility and flexibility provides unique advantages to formulate uniform and continuous nanofibers with controllable pore structure from many natural polymers and synthetic polymers [44,45,46]. The electrospun nanofibers with a large surface-to-volume ratio and high porosity have been used in various fields, especially biomedical applications such as tissue engineering, drug delivery, and wound dressing [44,47,48]. Nanofibers have been useful in tissue regeneration because they imitate the natural extracellular matrix (ECM) and promote the migration and proliferation of cells [49]. The properties of electrospun fiber that can benefit the arena of wound healing include high porosity, swelling capacity, good gaseous exchange, and excellent cellular adhesion, possess antimicrobial properties, and the capability to provide moisture for accelerated wound healing process [50,51]. Another exceptional advantage of the electrospinning technique that can be beneficial in the field of wound healing is that complex hierarchical structures can be gotten by controlled calcination [52]. These complex structures are not easy to prepare to utilize conventional approaches like self-assembly, template-assisted synthesis, and other solution-based procedures.

Although the electrospinning technique demonstrates interesting advantages over other techniques that are used for nanofiber fabrication, it also has some limitations [53]. First, in the fabrication of organic nanofibers, the diversity of polymers employed in electrospinning is limited, and the performance and structure of nanofibers are not well investigated. Second, the range and performance of the application of electrospun inorganic nanofibers have been restricted because of their friability post calcination, although inorganic nanofibers have a potential application in many areas such as biological tissue engineering and other fields [53]. Lastly, it remains challenging to formulate electrospun nanofibers with diameters less than 10 nm [54].

## 5. Fabrication of Biopolymer-Based Hybrid Nanofibers

### 5.1. Chitosan–PVA/PCL Hybrid Nanofibers

**Figure 4:**
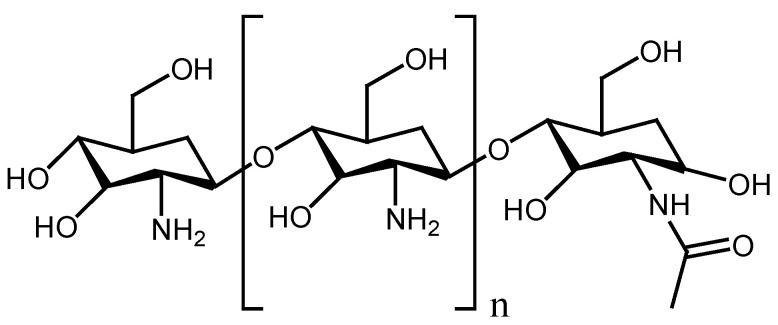
Molecular structure of chitosan.

Chitosan is a linear copolymer (Figure 4) composed of chitin, a principal constituent of the exoskeletons of crustacea such as crab and shrimp. It is a biopolymer that is well-known for its attractive properties such as its non-toxicity, inertness, biocompatibility, biodegradability, non-antigenicity, bio-adhesiveness, hemostatic properties, antimicrobial effects, antimicrobial properties, and wound healing features [55]. In addition, it is very versatile and can produce a diversity of functionalized derivatives via chemical modifications of hydroxyl and amino groups. Chitosan and its derivatives have also been reported to demonstrate mild gelation while exhibiting the capability to produce films. It displays strong attachment to lesion tissues and stimulates hemostasis and wound healing mechanisms [27]. There are many reports of crosslinked chitosan formulation of nanofibers for wound healing applications.

Ganesh et al. formulated chitosan–PVA electrospun composite nanofibers encapsulated with silver (Ag) nanoparticles and sulfanilamide for wound care [56]. The successful preparation of the nanofibers was confirmed by X-ray diffraction (XRD) and Fourier-transform infrared (FTIR) spectroscopy. Scanning electron microscopy (SEM) studies of hybrid nanofibers demonstrated smooth morphology (mimicking ECM) with good structural integrity with an average fiber diameter of approximately 95 nm that can improve cell proliferation and promote superior penetration of cells into the fibers. The swelling analysis of nanofibers showed a high swelling (200%) compared to the free PVA nanofibers patch and the drug-loaded nanofibers. The in vitro drug release studies at physiological conditions (pH 7.4 and 37 °C) demonstrated the initial burst release of the drug and nanoparticles followed by a slow and sustained release. The drug release profile promoted the constant antibacterial efficacy of the wound dressing in the wound environment. The in vivo wound healing analysis using wounds on rat model promoted fast wound contraction of approximately 90.76 ± 4.3% for dual drug-loaded nanofibers after seven days compared to 55.26 ± 3.5% wound reduction for the plain nanofiber after 20 days [56].

Adeli et al. prepared electrospun chitosan–PVA hybrid nanofibers for wound healing applications. The porosity analysis of nanofibers utilizing the liquid displacement technique displayed high porosity of more than 91%, which can be advantageous for wound respiration. The nanofibers exhibited tensile strength between 3 and 6 MPa and elongation at a break of approximately 160–180%, which are suitable for wound dressing and skin regeneration application. The water vapor transmission analysis of the electrospun nanofibers showed a water vapor transmission rate (WVTR) that ranged 2340–3318 g/m^2^·24 h revealing their ability to maintain appropriate moisture for the wound healing process. The in vitro biocompatibility studies displayed the cell viability that ranged between 72–95% when the nanofibers were incubated with mouse fibroblast cells (L929) for 24 h, confirming low toxicity. The in vitro antimicrobial analysis of electrospun chitosan–PVA nanofibers exhibited antibacterial activity that ranged between 60–84% and 47–72% for *Staphylococcus aureus* (*S. aureus*) and *Escherichia coli* strain (*E. coli*), respectively [57].

Iqbal et al. designed chitosan–PVA nanofibrous materials encapsulated with cefadroxil for bacteria-infected wounds. The drug release kinetics in vitro displayed a fast release of cefadroxil at the first hours, followed by a sustained drug release. The in vitro cytotoxicity studies using MTT assay showed high cell viability of about 92.47% ± 2.36 when the nanofibers were immersed with proliferating human epidermal keratinocytes. The in vitro antibacterial analysis demonstrated superior antibacterial efficacy against all the screened clinical *S. aureus* strains compared to the plain cefadroxil and nanofibers [58]. Alavarse et al. formulated electrospun nanofibers based on chitosan and PVA. They were loaded with tetracycline hydrochloride for wound dressing. The in vitro antimicrobial analysis showed high bacterial effects of antibiotic-loaded nanofibers against *E. coli*, *S. epidermidis*, and *S. aureus*. The wound healing analysis in vitro using scratched rabbit aortic smooth muscle cells exhibited a higher cell migration rate of approximately 8%/h for nanofibers and about 6%/h for control during the initial 12 h, revealing their potential to accelerate wound closure [59].

Zhou et al. prepared *N*-carboxyethyl chitosan–PVA nanofibers for wound management. The in vitro biocompatibility studies using MTT assay showed high cell viability of the L929 cells when immersed with nanofibers for 48 h, indicating the low toxicity of *N*-carboxyethyl chitosan–PVA nanofibers [60]. Majd et al. designed chitosan–PVA nanofibers for the management of diabetic wounds. The in vivo wound healing analysis of the nanofibers demonstrated a significantly accelerated healing process of diabetic wounds in the streptozotocin-induced diabetic rats treated by nanofiber [61]. Ibrahim et al. prepared carboxymethyl chitosan–PVA electrospun nanofibers loaded with gold (Au) nanoparticles. The in vitro cytotoxicity studies showed cell viability of 96.1 ± 0.3% when the nanoparticle-loaded nanofibers were incubated with epidermis cell line (A549). The in vitro antimicrobial experiments of the nanofibers demonstrated increased antibacterial efficacy when Au nanoparticles increased on Gram-negative and Gram-positive bacteria, which confirmed that the interaction of carboxymethyl chitosan with Au nanoparticles proceed through hydroxyl functional groups, not via the amine groups [62].

Bakhsheshi-Rad et al. synthesized nanofibers that are based on chitosan and PVA containing silk protein sericin for wound care. The in vitro antibacterial analysis showed that the polymeric nanofibers encapsulated with high sericin amount inhibited the growth of *E. coli* and *S. aureus* strain. The in vitro biocompatibility studies employing MTT assay exhibited high cell viability of L929 fibroblast cells in the presence of sericin-containing nanofibers that was in the range of 75–102% after 3 days and 86–118% after 7 days. The in vivo wound healing studies using the mice model showed faster-wound closure when the wounds were treated with sericin-loaded nanofibers compared with when dressed with pristine nanofibers, demonstrating their ability to provide healing [63]. Kharaghani et al. formulated PVA–chitosan nanofibers loaded Ag and Au nanoparticles. These nanofibers showed superior antibacterial activity in vitro against *E. coli* and *bacillus,* especially those loaded with Ag nanoparticles, as opposed to those loaded with Au nanoparticles [64]. Zhang et al. designed 3-dimensional (3D) layered nanofiber sponges from the combination of chitosan and PVA for wound healing applications. The in vitro blood coagulation studied demonstrated that blood completely coagulated in the presence of a 3D nanofiber sponge compared to the gauze (control). The in vivo wound healing experiments using full-thickness wound on mice models demonstrated that skin wound repair was faster with a small scar area when treated with nanofibers compared to the gauze [65].

Sannasimuthu et al. formulated chitosan–PVA nanofiber scaffolds encapsulated with *Arthrospira platensis* for wound treatment. The in vitro cytotoxicity analysis exhibited high cell viability of NIH-3T3 mouse embryonic fibroblast cells when incubated with nanofibers, indicating their non-toxicity. The in vitro wound healing studies demonstrated a potential wound healing mechanism in NIH-3T3 cells [66]. Yang et al. prepared electrospun chitosan–PVA nanofibers incorporated with graphene oxide for microbial infected wounds. The antibacterial analysis employing zone of inhibition tests of nanofibrous scaffolds exhibited good bactericidal efficacy against *E. coli* and *S. aureus* [67]. Wang et al. prepared electrospun chitosan–PVA nanofibrous material incorporated with Cu metal-organic frameworks for wound dressing application. The in vitro cytotoxicity experiment showed a high cell viability of more than 90% when the nanofibers were incubated with L929 fibroblast cells with excellent cell adhesion and proliferation. The wound healing experiments in vivo using full-thickness skin wounds in mice demonstrated wound closures of 89.3% and 90.7% for commercial chitosan wound dressing and pristine chitosan–PVA nanofibers on the 12th day, respectively. The wound closure for chitosan–PVA nanofibrous material incorporated with Cu metal-organic frameworks was 99.1% without red and uneven scars [68].

Shokrollahi et al. formulated nanofibrous scaffolds from carboxymethyl chitosan and PVA incorporated with chamomile for antioxidant or antibacterial wound dressing. The antioxidant activity analysis utilizing DPPH radical scavenging assay demonstrated higher antioxidant efficacy of approximately 38.01 ± 0.08% for chamomile-loaded nanofibers compared to 6.00 ± 0.18% antioxidant efficacy for the unloaded nanofibers, indicating that the presence of chamomile accelerated the inflammation phase by destroying ROS. The in vitro antibacterial analysis exhibited high growth inhibitory effect against *E. coli* and *S. aureus* [69]. Ahmed et al. formulated electrospun chitosan–PVA nanofibers loaded ZnO nanoparticles for wound dressing application. The in vitro antimicrobial analysis demonstrated a high zone of inhibitions for nanoparticle-incorporated nanofibers against *P. aeruginosa*, *S. aureus*, *B. subtilis,* and *E. coli*, were, 21.8 ± 1.5, 21.5 ± 0.5, 15.5 ± 0.8 and, 20.2 ± 1.0 mm respectively when compared to plain nanofiber which was 15.8 ± 1.0, 5.4 ± 0.5, 13.0 ± 0.7 and 14.1 ± 0.8 mm, respectively. The in vivo wound closure experiments utilizing subcutaneous wounds in diabetes-induced rabbits demonstrated almost complete wound closure for nanoparticle-loaded nanofibers on day 12. In contrast, the plain nanofiber showed only 80% wound closure [70].

Koosha et al. fabricated electrospun chitosan–PVA hydrogel nanofibers reinforced by halloysite nanotubes for skin regeneration application [71]. FTIR spectrum confirmed the successful crosslinking between chitosan and PVA. The SEM images of the nanofibers demonstrated uniform morphology without any beads and showed well-dispersed and distributed halloysite nanotubes throughout the nanofiber matrix. The swelling analysis revealed that the hydrogel nanofibers absorb water faster when compared to hydrogel films because of their high surface-to-volume ratio and porous structure. The water contact angle studies showed that the addition of nanotubes significantly decreased the water contact angle of nanofibers from 59.2 ± 7.3° to 45.8 ± 4.2° resulting in more hydrophilic nature of nanofibers. The in vitro biocompatibility tests utilizing normal human fibroblasts showed that the biocompatibility and cell attachment of the cells on nanofibers loaded with halloysite nanotubes was higher than nanofibers without halloysite nanotubes and control [71]. Naeimi and co-workers formulated chitosan–PVA nanofibers loaded with a plant extract called *Nepeta dschuparensis* and honey for wound healing application. The in vivo wound healing experiments using second-degree burn wound on animal model demonstrated that the wounds treated with nanofibers loaded with *Nepeta dschuparensis* and honey healed faster with a wound closure percentage of 59.85% ± 0.14 at day 21 when compared with the control group (40.45% ± 0.008), plain nanofiber (37.42% ± 0.008), and silver sulfadiazine (silver topical treatment for burn wounds) (44.28 ± 0.009) [72].

Sarhan et al. synthesized electrospun nanofibers loaded with honey from a combination of chitosan and PVA. The antibacterial experiments in vitro of the nanofibers showed a superior antibacterial efficacy against *S. aureus* when there was an increase of chitosan concentration within the nanofibers [73]. Abdelgawad et al. designed chitosan–PVA nanofibers encapsulated with Ag nanoparticles for microbial infected wounds. The in vitro drug release results demonstrated that the % cumulative Ag nanoparticles release from the nanofibers was rapid during the initial 60 min after incubation in the releasing medium and then increased gradually thereafter. The in vitro antimicrobial analysis of Ag nanoparticle-loaded nanofibers using viable cell-counting exhibited superior synergistic antibacterial effects against *E. coli* bacteria by blending chitosan and Ag nanoparticles compared to the plain chitosan–PVA nanofibers, indicating their potential as antibacterial wound dressings [74].

Li et al. formulated electrospun nanofibers from chitosan oligosaccharides and PVA. They were encapsulated with Ag nanoparticles using glutaraldehyde vapor as a crosslinking agent for wound treatment [75]. The SEM micrographs demonstrated that the chitosan–PVA nanofibers had a smooth morphology with a mean fiber diameter of 130–192 nm. The in vitro antibacterial tests showed that nanofibers loaded with Ag nanoparticles possessed high antibacterial efficacy against *S. aureus* and *E. coli* bacterial strains. In contrast, plain nanofibers did not show any antibacterial effects [75]. The in vivo wound healing experiments using full-thickness circular wounds on Sprague Dawley rats exhibited that Ag nanoparticle-loaded nanofibers demonstrated excellent wound closure compared with commercially available wound-plasts gauze, which was used as the references, and the wound healing was time-dependent. Similar nanofibers were prepared from chitosan oligosaccharide and PVA by Chen-wen Li et al. The nanofibers were loaded with Ag nanoparticles. It demonstrated good wound contraction in rats treated with the nanofibers significantly faster than the gauze and plain nanofibers [76].

Kang et al. prepared chitosan-coated PVA nanofibers for wound treatment. The histological assessments and mechanical performance analysis showed that the chitosan–PVA nanofibers are more effective as a wound healing accelerator in the early stages of the wound healing process when compared to the heat-treated PVA nanofibers [77]. Son et al. designed chitosan–PVA nanofibers loaded with Ag nitrate and titanium dioxide for antimicrobial wound dressings. These nanofibers exhibited antibacterial activities of 98 and 99% against *E. coli* and *S. aureus*, respectively, suggesting that these nanofibers can be very useful for the treatment of bacteria-infected wounds [78]. Chen and Huang formulated electrospun *N*-maleoyl-functional chitosan–PVA nanofibers encapsulated with antibiotic tetracycline hydrochloride for wound care. These nanofibers displayed excellent water stability, insignificant cytotoxicity, high water retention capability, and reductant-responsive functions. The in vitro antibacterial experiments of antibiotic-loaded nanofibers showed superior antibacterial effects revealing their capability as good antibacterial wound dressings [79]. Zhou et al. prepared carboxyethyl chitosan–PVA nanofibrous scaffolds for wound management. The in vitro cytotoxicity experiments using fibroblast L929 cells showed the non-toxic nature of the electrospun nanofiber. Cell culture analysis results revealed the cell proliferation and adhesion capability of the electrospun nanofibers, which is important for skin regeneration [80].

Wang et al. formulated chitosan–PVA electrospun nanofibers for wound management. The SEM results displayed a ratio of chitosan and PVA at 50/50, resulting in a nanofibrous material similar to biological tissues. These electrospun nanofibers possessed the potential to be used for the management of infected wounds and skin regeneration [81]. Sundaramurthi et al. prepared electrospun chitosan–PVA nanofibers for wound dressing application. The chitosan–PVA nanofibers, together with the topical administration of growth factor, R-Spondin 1 using full-thickness wounds on rats, demonstrated 98.6% wound closure after 14 days post-surgery [82]. Celebi et al. developed electrospun antimicrobial chitosan–PVA nanofibers encapsulated with Ag ion-incorporated hydroxyapatite nanoparticles. The in vitro antimicrobial analysis of nanofibers showed excellent bactericidal efficiency against *E. coli*, indicating the potential application of the nanofibers as ideal antibacterial wound dressings [83]. Yang et al. reported chitosan–PVA–graphene oxide nanofibers encapsulated with ciprofloxacin for antibacterial wound dressing. The in vitro antimicrobial analysis using the agar disk diffusion method exhibited significantly improved antibacterial efficacy against *E. coli*, *S. aureus*, and *B subtilis* after loading ciprofloxacin in the nanofibers with excellent cytocompatibility when incubated with Melanoma cells in vitro [84].

Gholipour-Kanani et al. formulated electrospun chitosan–PVA nanofibers for burn wound dressing. The in vivo wound healing analysis using rat demonstrated that the healing process of the burn wound dressed with chitosan–PVA nanofibers was rapid [85]. Kegere et al. fabricated chitosan–PVA composite electrospun nanofibers loaded with *Bidens pilosa* for the treatment of bacteria-infected wounds. The in vitro antimicrobial studies showed pristine chitosan–PVA nanofibers inhibited 75.9 and 86% growth of *E. coli* and *S. aureus*, respectively. In contrast, *Bidens pilosa*-loaded nanofibers inhibited 91% growth of *S. aureus*, indicating that the presence of *Bidens pilosa* enhanced the antibacterial effects of the nanofibers [86]. Levengood et al. developed chitosan–PVA nanofibers for skin regeneration application. The wound closure analysis in vivo employing wounds on BALb/c mice demonstrated that the diameter of the wounds treated with nanofibers was significantly decreased compared to the control [87].

He et al. formulated antimicrobial electrospun nanofibers with quaternized chitosan-graft-polyaniline and PCL for wound dressing [88]. The mechanical analysis demonstrated these nanofibers have appropriate stretchability that ranged between 81.7% and 48.1, which was close to that of human skin (75–60%). The antibacterial experiments in vitro showed that the chitosan–PCL hybrid nanofibers possessed an outstanding killing ratio of more than 90% for *E. coli* and *S. aureus,* indicating their excellent antibacterial properties for microbial infected injuries. The wound healing experiments employing the mouse full-thickness wounds defect model showed that the wounds treated with chitosan–PCL hybrid nanofibers resulted in a complete wound closure within 14 days compared to Tegaderm™ film (used as the reference) and plain chitosan–PCL hybrid nanofibers [88].

Afshar et al. formulated electrospun PLA-chitosan core-shell nanofibers loaded with curcumin using the coaxial electrospinning method. The SEM pictures exhibited a bead-free smooth surface of PLA-chitosan core-shell nanofibers with a mean diameter of approximately 671 ± 172 nm and broad diameter distribution. The TGA results suggested that although PLA content was less than chitosan in the nanofibers (20.7% for PLA and 39% for chitosan), the presence of PLA in the nanofibers significantly improved their mechanical properties. The in vitro drug release studies of curcumin from the PLA-CS core-shell nanofibers showed a two-stage release pattern; an initial burst drug release and then a sustained release, when the drug was incorporated in the core layer of nanofibers; hence it has potential applications in drug delivery and wound dressing [89]. Gholipour-Kanani et al. produced PCL–chitosan–PVA nanofibrous scaffolds for wound healing application. The wound healing studies in vivo using full-thickness cutting wounds and full-thickness round burn wound on male Sprague Dawley rats demonstrated much better wound healing performance for nanofibrous scaffolds compared to the control samples [90].

Yin and Xu prepared electrospun chitosan–PCL nanofiber scaffolds encapsulated with *Aloe vera*. The water vapor transmission studies of *Aloe vera*-loaded nanofibers demonstrated a water vapor transmission rate of approximately 2000 mL/m^2^/day, suitable for an ideal wound dressing. The in vitro antibacterial analysis of *Aloe vera*-loaded nanofibers demonstrated bactericidal efficacy against S. aureus, and the inhibition rates of 99.9%, suggesting that the nanofibers had good effects on inhibiting *S. aureus*. Furthermore, the inhibition rates of plain nanofibers and *Aloe vera*-loaded nanofibers against *E. coli* were 92.92% and 96.68%, respectively [91]. Zhou et al. formulated chitosan–PVA electrospun nanofibers loaded with nitric oxide for wound care. The in vivo wound healing experiments utilizing a full-thickness cutaneous wound model of mice showed wound closure of about 95.31% on the 14th day, significantly higher than the plain nanofibers [92]. Lemraski et al. prepared chitosan/polyvinyl alcohol/copper nanofibers via an electrospinning technique. In vivo studies of the wound dressings showed that the wound area was significantly decreased revealing the efficacy of chitosan in wound healing. The incorporation of copper nanoparticles in the wound dressings induced a significant decrease in the wound surface area on the third day after application in induced full-thickness open excision-type wound on male Wistar albino rats. The wound dressings also good antibacterial activity on *E. coli* and *P. aeruginosa*, *S. aureus* and *B. cereus* [93].

Chand et al. synthesized electrospun chitosan–PCL–HA bilayered scaffold for effective wound healing applications. The in vitro cytotoxicity analysis employing MTT assay showed better cell viability, indicating good biocompatibility of nanofibers for wound management [94]. Poornima and Korrapati developed chitosan–PCL composite nanofibrous materials for co-delivery of resveratrol and ferulic acid in wound healing application. The SEM micrographs of the dual drug-loaded nanofibers exhibited continuous, beadless, smooth, and randomly oriented fiber morphology with a mean diameter of 240 ± 50 nm. The wound healing assessments in vivo utilizing full-thickness excision wounds on the dorsal surface of the Wistar mice showed that the dual drug-loaded nanofiber scaffolds possessed a faster-wound contraction rate in comparison with that of plain nanofiber and control (saline) [95]. Jung et al. formulated chitosan nanoparticle–PCL nanofibrous scaffolds for wound dressing. The in vitro biocompatibility studies exhibited no cytotoxicity, and chitosan nanoparticles adsorbed by van der Waals forces were released into aqueous environments and then penetrated the rat fibroblast cells [96].

### 5.2. Gelatin–PVA/PCL Hybrid Nanofibers

Gelatin is a collagen derivative that is regularly utilized as a hydrogel for biomedical applications, typically due to its good biocompatibility and biodegradability in the physiological environment and its excellent processability [97]. Its low antigenicity makes it a well-established biopolymer for different biological applications. Gelatin has been used for wound dressings for the management of chronic wounds by many researchers [98]. Ardekani et al. prepared electrospun gelatin–PVA nanofibers encapsulated with *Zataria multiflora* (ZM) essential oil for wound care [99]. The successful preparation of electrospun gelatin–PVA nanofibers loaded ZM was physicochemically confirmed by FTIR and gas chromatography–mass spectrometry (GC–MS). The swelling experiments showed that the formulated electrospun hybrid nanofibers have an excellent capacity to absorb a high amount of water in the range of approximately 400–900%. The in vitro biocompatibility studies demonstrated high cell viability of mouse L929 cells suggesting their non-toxicity with good antibacterial properties because the in vitro antimicrobial analysis demonstrated that the nanofibers encapsulated with 10% by weight of ZM essential oil possessed excellent antibacterial activity by completely inhibiting the growth of *P. aeruginosa*, *Candida albicans* and *S. aureus*, after 24 h of incubation. These outcomes have revealed that gelatin–PVA nanofibers encapsulated with ZM essential oil are promising candidates to be used as potential wound dressings [99].

Ahlawat et al. designed gelatin–PVA hybrid nanofibers loaded with *Carica papaya* for application in wound treatment. Transmission electron microscopic (TEM) results showed that *Carica papaya* with a particle size of 25 ± 5.709 nm. Field emission scanning electron microscope (FESEM) images of the hybrid nanofibers displayed continuous, bead-free, and smooth morphology with an average fiber diameter of approximately 140 ± 20 nm. The in vitro cytotoxicity experiments using MTT assay demonstrated 80% cell viability of NIH 3T3 fibroblast cells when immersed with *Carica papaya*-loaded electrospun nanofibers in 12 h, indicating the good biocompatibility of nanofibers. The in vitro antibacterial analysis utilizing agar disc diffusion technique demonstrated that *Carica papaya*-encapsulated gelatin–PVA hybrid nanofibers possessed the more significant inhibition zone on agar plate against *S. aureus* and *E. coli* compared to plain pure gelatin–PVA hybrid nanofibers [100].

Alishahi et al. fabricated electrospun core-shell gelatin–PVA–chitosan nanofibers encapsulated with glucantime for the treatment of Leishmania wounds. The SEM results displayed the uniform nanofibers with bead-free morphology and an average fiber diameter of about 404 nm, while the transmission electron microscopy (TEM) analysis confirmed the core-shell structure of the hybrid nanofibers. The mechanical characterization of nanofibers demonstrated ultimate tensile strength of 7.11 ± 0.38 MPa, Young’s modulus of 253.41 ± 12.13 MPa, and elongation at a break of 3.02 ± 0.11%, indicating good mechanical performance for wound dressing application. The in vitro drug release profile at the physiological conditions demonstrated approximately 84% of the encapsulated drug was released from the nanofibers in the initial 2 h and then after 5 h. Almost all the encapsulated drug was released [101]. The anti-Leishmania analysis using the flow cytometry method showed that the pristine nanofibers possessed almost antileishmanial efficacy (4.4 ± 0.3% cell death), which was similar to the untreated leishmanial parasites (0.7 ± 0.0% death) as the negative reference. However, glucantime-encapsulated hybrid nanofibers displayed leishmanicidal activity in a dose-dependent mode and destroyed 73.8 ± 2.3%, and 79.6 ± 2.2% of parasites with 4 and 6-cm^2^ of glucantime-incorporated nanofiber, respectively, with no toxic effects on the human fibroblast cells. These results demonstrated gelatin–PVA–chitosan nanofibers encapsulated with glucantime as the potential scaffolds for treating Leishmania wounds [101].

Ajmal et al. formulated PCL–gelatin-based hybrid nanofibers encapsulated with quercetin and ciprofloxacin hydrochloride for application in wound treatment [102]. The water contact angle studies showed an average contact angle values for plain PCL–based nanofibers, PCL–gelatin hybrid nanofibers, and dual drug-loaded PCL–gelatin nanofibers were 100.1 ± 3.16°, 55.5 ± 2.10°, and 48.8 ± 2.95°, respectively, confirming the hydrophilic nature of the nanofibers. The in vitro drug release profile demonstrated that the release of the loaded bioactive drugs from polymeric hybrid nanofibers was biphasic, with initial rapid drug release followed by slowly sustained release. The in vivo wound healing experiments demonstrated that wounds treated with dual drug-loaded PCL–gelatin nanofibers offered completely wound closure (100%) on the 16th day while wound closure by ciprofloxacin-loaded PCL–gelatin nanofibers, plain PCL–gelatin nanofibers, and gauze was 89.08%, 78.85, and 71.32, respectively [102].

Ghaee et al. fabricated PEG methyl ether methacrylate (PEGMA)-surface-modified PCL nanofibers within gelatin-chitosan hydrogels loaded with curcumin for application in skin regeneration. The porosimetry experiments exhibited suitable porosity of scaffolds for skin regeneration from 90.43 to 71.48% and pore size of approximately 101–256 μm. The biological studies confirmed the appropriate cell attachment and biocompatibility of the scaffolds. Furthermore, the antioxidant analysis of curcumin-loaded nanofibers in vitro displayed good antioxidant efficacy [103]. Shi et al. formulated gelatin–PCL–based electrospun nanofibers encapsulated trimethoxysilylpropyl octadecyl dimethyl ammonium chloride (QAS) (used as an antibiotic) for antibacterial wound dressing. The mechanical performance analysis of nanofibers showed that the tensile strength in the range of 9–12 MPa fulfills the clinical requirements of 1–2 MPa. The in vitro antibacterial experiments of antibiotic-loaded nanofibers displayed more than 99% bactericidal activity against *P. aeruginosa* and *S. aureus*, suggesting their efficacy as excellent antibacterial wound dressings [104].

Jafari et al. formulated bilayered nanofibers based on PCL and gelatin loaded with amoxicillin and zinc (Zn) nanoparticles for bacterial wound treatment [104]. The SEM images of PCL–gelatin nanofibers displayed a beadless and fine fibrous morphology with an average diameter of approximately 576.36 ± 197.77 nm. The swelling experiments of nanofibers demonstrated a high swelling degree of 196.8%, which may be due to hydrophilic gelatin. The incorporation of 2% by weight of Zn nanoparticles increased the swelling degree of the nanofibers to 228.5%, while amoxicillin decreased the swelling. The in vitro degradation studies showed that all the nanofibers had degradation rates between 37 and 53% after three weeks. The in vitro drug release assessments demonstrated that hybrid nanofibers have a sustained release manner for Zn nanoparticles and amoxicillin up to 144 h. The antibacterial analysis of the dual drug-loaded hybrid nanofibers in vitro employing the disk diffusion method exhibited the larger inhibition zone size between 13.42 ± 0.65 and 17.32 ± 0.42 mm against *S. aureus,* indicating good antibacterial efficacy. The wound healing experiments using circular full-thickness wounds on Sprague-Dawley rats revealed that the wound contraction percentage for the control was 64.77 ± 3.35, and 95.07 ± 1.51% on days 6 and 10 respectively, while dual drug-loaded hybrid nanofibers displayed a wound contraction percentage of 69.44 ± 3.65, and 95.60 ± 2.99% at the same time, respectively [105]. Basara et al. produced electrospun gelatin–PCL wound dressing nanofibers for controlled drug release of ketoprofen, an anti-inflammatory drug. The in vitro cytotoxicity experiments of the nanofibers using MTT assay displayed steadily increased cell viability of the L929 mouse fibroblast cells revealing no indication of toxicity. The in vitro drug release kinetics demonstrated sustained drug release of the ketoprofen from nanofibers; specifically, the binary electrospun nanofibers extended the ketoprofen release for approximately 4 days. This result points out that these nanofibers are much more efficient in retaining the drug and releasing it in a controlled phenomenon [106]. Naseri-Nosar et al. formulated electrospun gelatin–PCL nanofibers incorporated with cerium oxide nanoparticles as effective wound dressing scaffolds. The water uptake studies and water vapor transmission rate experiments showed that PCL–gelatin nanofibers had the water-uptake capacity and water vapor transmission rate of 15.80 ± 0.66% and 3608 ± 170.27 g·m^−2^, which can lead to the accelerated wound healing process. The wound healing studies in vivo using full-thickness excisional wound model on the male Wistar rats demonstrated that the cerium oxide nanoparticle-loaded nanofibers had the wound contraction of approximately 94.40 ± 0.63% and 98.80 ± 1.07% in 7 and 14 days after surgery, respectively. These values of wound closure were 15.51 ± 3.82% and 63.19 ± 8.86% for the gauze at the end of the same day, respectively. The percentages of wound closure of the nanoparticles-loaded nanofibers were higher than those of the gauze after 7 days [107].

Pavliňáková et al. designed electrospun nanofibers from gelatin and PCL reinforced with halloysite nanotubes. The in vitro cytotoxicity experiments of nanofibers exhibited cytotoxic based on the interaction with mouse fibroblasts (NIH-3T3 cells), suggesting that these nanofibers are suitable for wound dressing applications [108]. Farzamfar et al. formulated gelatin–PCL nanofibers encapsulated with Taurine for wound treatment. The SEM micrographs of nanofibers showed oriented random, dispersive manner, forming a non-woven porous morphology with average pore diameters of about 20.25 ± 3.52 µm and fiber diameter of 568.50 ± 135.62 nm. The water vapor transmission studies of the nanofibers showed a water vapor transmission rate of 3048.00 ± 209.23 g m^−2^, which is suitable for proper wound healing. The in vivo wound closure experiment employing full-thickness excisional wounds on adult male Wistar rats exhibited 92% wound closure when treated with taurine-loaded nanofibers compared to the sterile gauze (reference), which revealed approximately 68% of wound closure after 14 days [109].

Unalan et al. formulated electrospun gelatin–PCL nanofibers encapsulated with clove essential oil. These nanofibers demonstrated superior bactericidal efficacy in vitro against *E. coli* and *S. aureus* as potential antibiotic-free candidates for wound healing [110]. Ramalingam et al. prepared gelatin–PCL hybrid electrospun composite nanofibrous scaffolds loaded with a plant extract called *Gymnema sylvestre* for antibacterial wound dressing. The in vitro drug release studies demonstrated an initial burst release of the plant extract from the nanofibers that can contribute to the prevention of bacterial colonization [111]. Fu et al. fabricated gelatin–PCL nanofiber scaffolds enriched with human urine-derived stem cells for wound healing by stimulating angiogenesis. The in vivo wound healing experiments using full-thickness wounds in rabbits demonstrated that stem cell-loaded nanofibers significantly enhanced wound healing when compared to wounds dressed with pure gelatin–PCL nanofibers or undressed wounds. The gelatin–PCL nanofiber-dressed wounds closed much faster, with increased reepithelialization, angiogenesis, and collagen development [112].

Adeli-Sardou et al. formulated electrospun gelatin–PCL nanofibers encapsulated with lawsone for skin regeneration application. The mechanical characterization of 1.5 wt% lawsone-loaded nanofibers displayed tensile strength (MPa), strain at break (%), Modulus (MPa) values 0.765 ± 0.06 MPa. 10.7 ± 1.5%, and 0.6 ± 0.61 MPa, respectively. The in vitro antimicrobial analysis of nanofibers using the disc diffusion method showed that the antibacterial activity against *S. aureus* and *P. aeruginosa* increased as the content of lawsone increased in the gelatin–PCL nanofibers while the pristine gelatin–PCL nanofibers did not display any bactericidal properties. The in vivo wound closure studies using circular wounds that were made on the posterior of the rats showed that the whole wound area was closed significantly when dressed with 1.0 wt% lawsone-loaded nanofibers and 1.5 wt% lawsone-loaded nanofibers by 96.3 ± 4% and 100% closure after 14 days, respectively; while the wound closure was 71.4 ± 2% for plain gelatin–PCL nanofibers [113].

### 5.3. Alginate–PVA/PCL Hybrid Nanofibers

**Figure 5:**
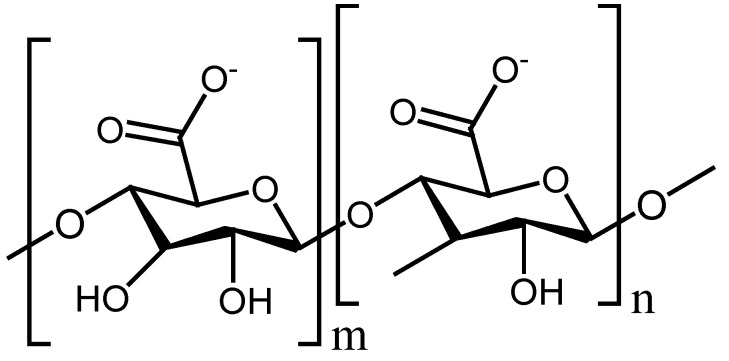
Chemical structure of alginate.

Alginate, also known as alginic acid (Figure 5), is one of the most applied and studied polysaccharides in drug delivery and tissue engineering applications. Alginate has also been well-recognized as wound-healing material and has high biocompatibility, low toxicity, biodegradability, and good mucoadhesive features [114]. Najafiasl et al. fabricated electrospun core/shell hybrid nanofibers that are based on sodium alginate and PVA. They were loaded with dexpanthenol for wound treatment. The SEM images of nanofibers demonstrated uniformity and beadless morphology with a mean diameter that reduced from 130.54 nm to approximately 100 nm because of the use of the shell in the nanofiber structure. The in vitro drug release results exhibited that the presence of 1% by weight of chitosan in the hybrid nanofibers assisted better control of the drug release of dexpanthenol. The in vitro cytotoxicity experiments using MTT assay and cell culture revealed that dexpanthenol-encapsulated sodium alginate–PVA nanofibers possessed a non-toxic effect on the fibroblast cells with appropriate cellular adhesion and morphology. All the above results indicated that dexpanthenol-encapsulated sodium alginate–PVA nanofibers are suitable scaffolds for wound healing applications [115].

Zhu et al. prepared alginate–chitosan–PVA coaxially electrospinning nanofibers loaded with asiaticoside for the treatment of deep partial-thickness burn wounds. The SEM micrographs of asiaticoside-loaded coaxial nanofibers exhibited smooth morphology, uniform distribution, few beads, and larger diameter than plain coaxial nanofibers, due to the low conductivities and viscosities of the asiaticoside-loaded polymer solution. The in vitro drug release kinetics of coaxial nanofibers in physiological conditions displayed accelerated drug release rates and high asiaticoside release compared to the *Centella* triterpenes cream, which accelerated the drug release to facilitate the wound healing process. The in vivo burn wound healing experiments using rats showed high wound closure at day 21 of the asiaticoside-loaded nanofibers was 99.2% ± 1.11, suggesting a good wound healing mechanism of nanofibers. Furthermore, Asiaticoside-loaded coaxial hybrid nanofibers offer a novel opportunity for the management of deep partial-thickness burn wounds [116].

Shalumon et al. formulated sodium alginate–PVA electrospun nanofibers enriched with ZnO nanoparticles for microbial infected wound treatment. The cell adhesion and spreading experiments using L929 cells demonstrated that the cells adhered very well to the hybrid nanofibers in 2 days. However, it was toxic and affected by an increased amount of ZnO nanoparticles and display excellent spreading after 4 days. Lastly, the in vitro antimicrobial studies utilizing diffusion disk tests showed the antibacterial activity (inhibition zone) of nanofibers against *E. coli* and *S. aureus* increased as the concentration of ZnO nanoparticles increased from 0.5 to 5% by weight in 24 h incubation. These results suggested that the ZnO nanoparticle-loaded polymeric hybrid nanofibers are promising scaffolds for application as wound dressings with a considerable concentration of ZnO nanoparticles [117].

Arthanari et al. formulated gatifloxacin-incorporated alginate–PVA electrospun nanofibers. The XRD and FTIR spectrums displayed excellent interactions between sodium alginate and PVA, probably caused by hydrogen bonds. The in vitro drug release experiments demonstrated controlled and continuous release of the gatifloxacin from the nanofibers within the first 6 h [118]. Fu et al. formulated electrospun sodium alginate–PVA nanofiber scaffolds loaded with moxifloxacin hydrochloride for microbial infected wounds. The SEM micrographs of antibiotic-loaded nanofibers displayed uniform fibrous structures on the morphology with a fiber diameter of about 175 ± 75 nm. The swelling analysis of hybrid nanofibers showed a swelling degree of 108 ± 6.45%, suggesting the ability of nanofibers to provide a moist environment for the acceleration of the healing process. The drug release studies in vitro demonstrated the initial burst release stage in the first 8 h and then followed by a gradual increase in the cumulative release until a plateau was reached at 26 h. The antimicrobial studies of antibiotic-loaded nanofibers showed a high zone of inhibition against *S. aureus* and *P. aeruginosa* revealing good antibacterial efficacy of nanofibers. The wound closure studies in vivo in the full-thickness round wounds on the back of rats demonstrated a superior and faster healing mechanism for antibiotic-loaded fibers than the blank nanofibers and untreated wounds [119].

Rashtchian et al. prepared biaxial electrospun hybrid nanofibers loaded with nanocrystal cellulose from alginate and PCL. The cytotoxicity analysis of nanofibers in vitro showed high cell viability of fibroblast cell line NIH/3T3 of more than 90 %, indicating a non-toxic effect of nanofiber. The SEM images of the fibroblasts on the hybrid nanofiber scaffolds displayed better cell attachment on alginate–PCL nanofibers loaded with nanocrystal cellulose, confirming excellent non-toxicity and biocompatibility for skin wound healing [120]. Udaseen et al. fabricated electrospun hybrid nanofibers from alginate and PVA. The SEM micrographs of nanofibers showed morphology with small beads with an average diameter ranging between 100 and 400 nm that can be very useful in wound dressing [121]. Coskun et al. formulated sodium alginate–PVA nanofibers for wound dressing. The in vivo wound healing studies using wound on rabbit model. The nanofibers demonstrated superior wound healing performance (i.e., epidermis characterizations, epithelization, vascularization, and hair follicle development) [122]. Üstündağ et al. formulated sodium alginate–PVA electrospun nanofibrous scaffolds for wound care. The SEM analysis of nanofibers showed bead-free morphology imitating ECM with an average fiber diameter of about 100.35 ± 12.79 nm. The in vivo wound healing experiments demonstrated complete wound closure at day 21 of dressing with nanofibers in comparison with gauze, which was used as control [123].

### 5.4. Cellulose–PVA/PCL Hybrid Nanofibers

**Figure 6:**
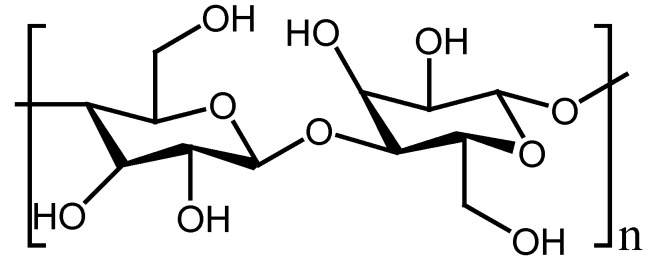
Chemical structure of cellulose.

Cellulose is the major structural constituent of plant cell walls and is the most abundant biopolymer. It is a renewable biomaterial readily accessible and affordable. Cellulose is a linear biopolymer established by β-1,4 linked D-glucose units which are joined to form cellobiose repeating units (Figure 6) [124]. The wound healing efficacy of cellulose has been reported in several studies, which reveal that this polymer speeds up the wound healing process through the release and maintenance of various growth factors at the site of injuries, such as epidermal growth factor (EGF), phosphodiesterase growth factor, and basic fibroblast growth factor (bFGF) [125]. Shef et al. formulated curcumin-loaded hydrogel nanofibers based on cellulose and PVA for application in wound treatment. The in vitro biocompatibility studies of curcumin-loaded hydrogel nanofibers showed almost 100% cell viability of L929 cells, confirming non-toxicity.

In contrast, the cell proliferation analysis in L929 cells exhibited increased cell proliferation when incubated with hydrogel nanofibers with a period that ranged between 1 and 7 days. The in vitro drug release studies displayed that the release of curcumin from the nanofiber in the physiological conditions was dramatically increased after 1 day of incubation. The in vivo wound healing experiments using full-thickness excision wounds in a rat model demonstrated 81.3 ± 1.3%, 50.63 ± 1.12%, 35.63 ± 1.3% wound closure for wounds treated with curcumin-loaded hydrogel nanofibers, plain hydrogel nanofibers, and untreated wounds (used as the reference) after two weeks of surgery, showing good wound healing properties and other biological effects of curcumin [126].

Zulkifli et al. formulated hydroxyethyl cellulose–PVA nanofiber scaffolds for skin regeneration. The SEM micrographs demonstrated uniform, beadless, porous morphology with an average fiber diameter of 20–373 nm. The contact angle analysis of the hydroxyethyl cellulose–PVA nanofiber scaffold showed a water contact angle of about 31.9°, suggesting the hydrophilic nature of scaffolds. The mechanical characterization of the nanofibers displayed the tensile strength of about 2.32 MPa, the elastic modulus of 97.8 MPa, and elongation at a break of approximately 12.43%. The SEM analysis displayed better cell proliferation and attachment and attachment on hydroxyethyl cellulose–PVA scaffolds with high cell viability of human fibroblast cells after seven days of culture, thus, supporting the potential of nanofibers as promising scaffolds for skin engineering applications [127].

Ahmed et al. fabricated cellulose acetate–PCL nanofibers incorporated with metallic nanoparticles (Ag, CuO, and ZnO nanoparticles) for wound disinfection applications. The FTIR and XRD data confirmed the successful fabrication of metallic-loaded nanofibers. The in vitro antimicrobial analysis of metallic nanoparticle-loaded nanofibers utilizing agar disc diffusion assay showed a zone of inhibition that was around 8.2 ± 0.9 mm against *E. coli*, while it was about 9.2 ± 1.6 mm against *S. aureus*, suggesting good antibacterial for treatment of microbial infected wounds [128].

Khoshnevisan et al. formulated cellulose acetate–PCL nanofibrous scaffolds enriched with propolis for wound healing application. These nanofibers displayed a very high significant water absorption capacity of approximately 400%. The in vitro antioxidant analysis of Propolis-loaded nanofibers exhibited excellent antioxidant efficacy that can contribute to the acceleration of the inflammation phase. The in vitro antibacterial studies showed that pure nanofibrous scaffolds possessed no bacterial inhibition activity. At the same time, Propolis-loaded nanofibers exhibited superior bactericidal effects against *S. epidermidis* and *S. aureus* [129].

### 5.5. Hyaluronic Acid–PVA/PCL Hybrid Nanofibers

**Figure 7:**
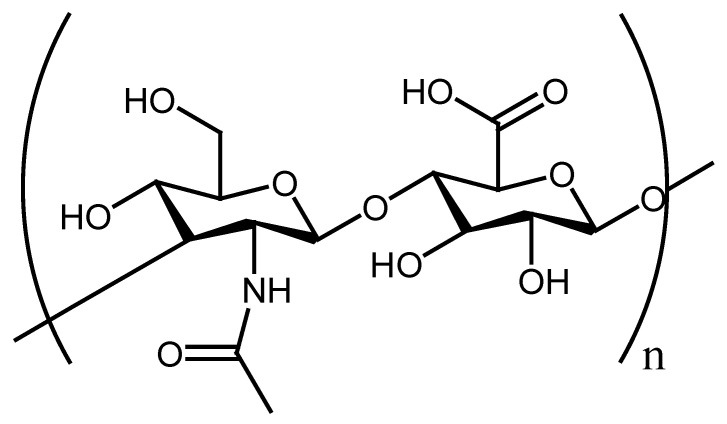
Chemical structure of hyaluronic acid.

Hyaluronic acid (HA) is a polysaccharide with a diversity of biological properties and wound healing potential. It is composed of disaccharide units comprising of *N*-acetyl-glucosamine and glucuronic acid (Figure 7). HA is normally extracted from the vitreous humor, umbilical cord, synovial fluid, or from rooster combs [130]. It is a non-toxic, non-allergic, and biocompatible biopolymer with a broad range of biomedical applications. It is also well-recognized as effective biomaterials for wound dressing applications. Several studies have demonstrated the wound healing mechanism of HA to promote epithelial and mesenchymal cell differentiation and migration, thus enhancing blood vessel development and collagen deposition [131]. Séon-Lutz et al. reported HA–PVA nanofibers prepared from hydroxypropyl-βcyclodextrin (HP βCD) for the formation of uniform nanofibers. These nanofibers displayed surface tension that ranged between 45 and 47 Nm^−1^. The SEM pictures of the HA–PVA nanofibers showed a smooth and uniform surface with an average diameter of 156 ± 31 nm. The in vitro cytotoxicity studies demonstrated high cell viability of NIH3T3 fibroblast cells when immersed with HA–PVA nanofibers for 24 h. The swelling analysis of the nanofibers displayed a significant swelling that ranged between 60% and 70% of the relative humidity that can provide suitable moisture for wound healing acceleration. The in vitro drug release kinetics of nanofiber at 33 °C (mean temperature of the skin) displayed a slight burst release of naproxen that can contribute to the rapid wound healing process, specifically the inflammatory phase [132].

Chen et al. fabricated maleilated hyaluronate-methacrylated PVA nanofibers for wound dressing. The XRD analysis confirmed the successful fabrication of polymeric hybrid nanofibers. The liquid uptake studies showed that these nanofibers exhibited high water absorption capacity over a prolonged period indicating their suitable application as ideal wound dressings. The in vitro cytotoxicity analysis of nanofibers using MTT assay showed high cell viability of L929 cells. The cell adhesion studies showed that L929 cells attached to the nanofiber surface and revealed a healthy and fibroblast-like morphology [133].

Wang et al. prepared hyaluronan–PCL electrospun nanofibers encapsulated with epidermal growth factors. The SEM images of the nanofibers showed porous and uniform fibers with a fiber diameter of 149 ± 4.5 nm and pore size of 0.17 ± 0.03 mm^2^. The in vivo wound healing studies using full-thickness skin defect wounds with a size of 18 mm × 18 mm on the dorsum of rats demonstrated a complete epidermis that was observed in the wound site after 4 weeks, whereas the thickest epidermis could be seen in the growth factor-loaded hybrid nanofibers treatments, such as hair follicle could only be seen in the growth factor-loaded hybrid nanofibers [134].

### 5.6. Collagen–PVA/PCL Hybrid Nanofibers

Collagen is the most naturally rich protein of the ECMs present in human tissues (e.g., cartilage, tendon, bone, skin, ligaments, etc.). It comprises 25% of the whole protein human body content [135], giving integrity and strength to tissue matrices [136]. Various recent studies have demonstrated the in vivo and clinical activity of collagen-based wound dressing material for accelerated granular tissue development, enhancing the healing rate and angiogenesis as well as the prevention of bacterial infection in chronic wounds [137]. Senthil et al. formulated hybrid nanofibers from collagen and PVA. Graphene was loaded into the fibers for wound healing application. The in vitro cytotoxicity studies of nanofiber showed that the graphene oxide-loaded nanofibers possessed a 100% cell viability and higher proliferation rate of HaCaT cells when compared to plain nanofibers. The wound closure studies in vivo further revealed that the nanofibers loaded with graphene oxide promoted 100% complete wound healing after day 12, whereas wound closure of the reference was only 20%, PVA nanofibers was 45%, and plain hybrid nanofiber was 80% [138].

Sobhanian et al. fabricated electrospun collagen-grafted PVA–gelatin-alginate nanofibrous scaffolds for skin regeneration. The mechanical characterization of collagen-grafted PVA–gelatin-alginate nanofibers displayed ultimate tensile strength, Young’s modulus, and elongation at a break of 4.33 ± 0.44 MPa, 150 ± 50 MPa, 3.41 ± 0.67%, respectively. The swelling behavior experiments showed a significant-high swelling degree of about 624.08 ± 110% and 801.25 ± 41% for 1 and 24 h, respectively, suggesting good swelling behavior for cell attachment. The water vapor transmission studies showed a water vapor transmission rate of 1575.72 g m^−2^ day^−1^, which was suitable to provide good moisture for accelerating the wound healing process. The cytocompatibility studies in vitro employing Indirect MTT assay showed significant-good cell viability of L929 cells with high cell proliferation [139]. Fu and Wang formulated Collagen–PCL nanofiber scaffolds for wound care. The results from this study revealed that human adipose stromal cells grown on the nanofibers have higher proliferation and migration rate, elongated morphology, and higher formation of ECM components, all of which happen during the process of wound healing [140]. Hou et al. formulated collagen–PCL electrospun nanofibers loaded with an antioxidant agent called *N-*acetylcysteine for application in wound management. The in vitro drug release experiments at physiological conditions showed that the *N-*acetylcysteine was initially a burst release from the nanofibers in 24 h followed by a slow and sustained drug release for post 24 h. The in vivo wound healing study of nanofibers was performed using rat wound model, and the percentage of the closure of wound area for the *N*-acetylcysteine-loaded nanofibers was 84.34% and 93.59% on day 9 and day 12, respectively, in comparison with the PCL nanofibers and pristine hybrid nanofibers, which displayed a wound closure of about 53.49% and 75.84% on day 9, 57.44% and 85.05% on day 12, respectively [141].

Tort et al. designed three-layered coaxial electrospun nanofibers from collagen and PCL layered with chitosan and alginate. The nanofibers were loaded with doxycycline for wound dressing. The water contact angle analysis showed a contact angle value of 38°, indicating excellent hydrophilic nature and wettability of nanofibers. The in vitro cytotoxicity studies of three-layered coaxial electrospun nanofibers loaded with doxycycline using keratinocyte (HaCaT) and fibroblast cell lines revealed good biocompatibility [142].

### 5.7. Gum Arabic/Gum Tragacanth–PVA/PCL Hybrid Nanofibers

Gum tragacanth is one of the most broadly utilized natural gums, which has found applications in many biomedical fields. Some of its attractive properties are readily available, biodegradability, biocompatibility, non-toxic nature, long shelf-life properties, and high resistance to bacterial invasion. Gum tragacanth and Gum Arabic have been employed for application in skin regeneration, wound dressing, and drug delivery applications [143]. Ranjbar-Mohammadi et al. prepared novel polymeric nanofibers based on gum tragacanth and PVA for the treatment of bacteria-infected wounds [144]. The FTIR spectra of the nanofibers confirmed their successful preparation by showing the peaks of the expected functional groups. The in vitro cytotoxicity and cell adhesion analysis demonstrated excellent cell viability when the nanofibers were immersed with human fibroblast cells and good cell attachment, proliferation, and growth on the nanofibers. The MTT assay displayed that the *p*-value for gum tragacanth–PVA nanofibers was more than 0.05, suggesting that these nanofibers are appropriate for tissue regeneration applications. The antibacterial studies of gum tragacanth–PVA employing disk shape webs demonstrated good antibacterial activities against *S. aureus* and *P. aeruginosa*. These results showed that these electrospun nanofibers are potential wound dressing material. They can protect the injury from its surroundings to prevent infection and dehydration and accelerate the wound healing process by removing any excess wound exudates, offering an optimum microenvironment for healing, and permitting continuous skin regeneration [144].

Ranjbar-Mohammadi et al. fabricated gum tragacanth–PVA nanofibers loaded with curcumin. The in vitro cell proliferation analysis demonstrated that the curcumin-loaded nanofibers possessed increased cell proliferation of fibroblasts with an increasing culture period from 1 to 3 days. Studies of cell metabolism showed that the nanofibers loaded with small amounts of curcumin significantly promoted cell adhesion and proliferation compared to the nanofibers loaded with high amounts of curcumin [145]. Eghbalifam et al. prepared PCL–coated gum arabic–PVA nanofibers incorporated with Ag nanoparticles for antibacterial wound dressing. The SEM results confirmed the presence of Ag nanoparticles in nanofibers, and the average fiber diameter of the polymeric nanofibers ranged between 150 and 250 nm with the porosity, water vapor permeability, and water absorption of 37.34%, 2235.50 g/m^2^.day, and 547.30%, respectively, revealing acceptable parameters for skin regeneration. The in vitro cytotoxicity analysis using MTT assay demonstrated that these nanofibers could support cell growth; although, cell proliferation was decreased by a high content of Ag nanoparticles. The in vitro antimicrobial experiments utilizing the agar diffusion assessment showed excellent antimicrobial efficacy for Ag nanoparticle-loaded nanofibers against three bacterial strains, *P. aeruginosa*, *E. coli*, and *S. aureus*, and a fungal strain, *C. albicans* (yeast) by a high inhibition zone [146].

Mohammadi et al. formulated gum tragacanth–PCL–PVA nanofibers for the management of diabetic wounds. The mechanical analysis of the nanofibers demonstrated a tensile strength of 2.7 MPa and a Young modulus of 56 MPa. The water contact angle measurements of gum tragacanth–PCL–PVA nanofibrous scaffolds were approximately 74 ± 2°, indicating the hydrophilic nature of nanofibers useful in cell proliferation and adhesion. The wound healing studies of gum tragacanth–PCL–PVA nanofibers in vivo using diabetic male Wistar rats demonstrated faster-wound contraction with complete wound closure on the 15th day compared to the gum tragacanth–PCL [147].

Ranjbar-Mohammadi and Bahrami formulated gum tragacanth–PCL nanofibers loaded with curcumin for biomedical application in wound healing. The mechanical characterization of nanofibers displayed tensile strength, elastic modulus, and strain at a maximum of 1.23 ± 0.10 MPa, 2.13 ± 0.10 MPa, and 87.00%, respectively. The in vitro biodegradation experiments showed that the few fibers in curcumin-loaded nanofibers were broken down after the 15th day of incubating them in phosphate buffer saline solution. The in vitro drug release studies displayed an initial burst release of curcumin from nanofibers followed by a sustained drug release up to 3 weeks that can contribute to the fast healing process of diabetic wounds [148]. Ranjbar-Mohammadi fabricated electrospun gum tragacanth–PCL nanofibers loaded with a plant extract, *Aloe vera*, for wound management. The SEM micrographs displayed that increasing the content of *Aloe vera* in the nanofibers significantly decreased the average diameter from 184 ± 34 to 123 ± 22 nm. The in vitro cell proliferation of *Aloe vera*-loaded nanofibers using MTT assay showed more proliferation of fibroblasts than the plain nanofibers [149].

Ranjbar-Mohammadi et al. formulated gum tragacanth–PCL nanofibers loaded with curcumin for bacterial infected and diabetic wound care. The in vitro antibacterial analysis of curcumin-loaded gum tragacanth–PCL nanofibers showed 99.9% and 85.14% antibacterial efficacy against MRSA and ESBL, respectively, revealing that these nanofibers are very promising candidates for antimicrobial applications. The in vivo wound healing studies using diabetic wounds on male Sprague Dawley rats demonstrated that the wounds treated with curcumin-loaded nanofibers showed accelerated wound healing by 100% wound closure on day 15, and the control wound area decreased by 20.96 ± 1.35% [150].

### 5.8. Silk Fibroin–PVA/PCL Hybrid Nanofibers

Silk fibroin, a natural protein polymer, has increasingly gained research interest as a promising biomaterial in drug delivery applications due to its excellent biological and physicochemical properties. Owing to its slow biodegradability, biocompatibility, and hemocompatibility [151], it has been used in various biomedical applications. It has been evident that silk fibroin promotes epidermal cells and fibroblast attachment, cellular proliferation, granular tissue formation and thus stimulates wound healing processes [152]. Huang et al. formulated silk fibroin–PVA nanofibers enriched with epidermal cells differentiated from human exfoliated deciduous teeth stem cells for wound repair. The SEM images demonstrated a uniform, beadless morphology (mimicking ECM) with interconnected pores with an average fiber diameter of 300 ± 180 nm. The in vivo wound healing experiments using 8 mm diameter circular full-thickness wounds with were on the backs of mice demonstrated restoration of granulation tissue on day 3, and the wound closure was 53.49% [153]. Kheradvar et al. prepared silk fibroin–PVA–*Aloe vera* loaded with starch nanoparticles used as a vitamin E-TPGS carrier. The starch nanoparticles encapsulation efficiency in nanofibers was about 91.63%, indicating that almost all the amount of starch nanoparticles were successfully encapsulated into the nanofibers. The water contact angle of the nanofiber was 38.23 ± 1.9° with a water uptake of approximately 130 ± 5.4%. The in vitro drug release profile showed an initial rapid release of starch nanoparticles from the nanofibers followed by a slow, sustained drug release. The in vitro antioxidant analysis showed that the nanofibers exhibited a DDPH scavenging effect. Increasing the starch nanoparticle content resulted in a higher antioxidant activity which could have a positive effect on the wound-healing process by protecting the cells from toxic oxidation products [154].

Ojah et al. formulated core-shell silk fibroin–PVA nanofibers loaded with an antibiotic, amoxicillin trihydrate, for wound dressing applications. The FTIR and XRD spectrums confirmed the successful formulation of antibiotic-loaded nanofibers. The mechanical performance studies of the nanofibers loaded with amoxicillin showed a tensile strength of 5.0 ± 0.2 MPa, Young’s modulus of 12.0 ± 1.4 MPa, and elongation at break of 32%. The in vitro antibacterial studies of nanofibers demonstrated excellent antibacterial activity by showing a high zone of inhibition against *E. coli* and *S. aureus* [155]. The antibacterial results of silk fibroin–PCL nanofibers formulated by Li et al. demonstrated high antibacterial activity of approximately 95% against *E. coli* and *S. aureus*. The results revealed good bactericidal effects of the nanofibers for the management of microbial infected wounds [156]. The silk fibroin–PVA hybrid nanofibers formulated by Chouhan showed accelerated wound healing of diabetic wounds on rabbit models with rapid granulation tissue formation, faster angiogenesis, and reepithelialization of wounds. These findings suggest that silk fibroin-based hybrid nanofibers can regulate ECM deposition resulting in rapid and complete repairs of chronic diabetic wounds [157].

### 5.9. Lignin–PVA/PCL Hybrid Nanofibers

Lignin is an amorphous phenolic biopolymer that is randomly branched and crosslinked with hemicellulose and cellulose. It is extracted from biomass through different processes, and the isolated one is called technical lignin. Due to lignin’s high mechanical performance, the use of lignin in wound dressings assists in the protection of the lesion from further injury or impurity [158]. Aadil et al. fabricated electrospun hybrid nanofibers based on lignin and PVA incorporated with Ag nanoparticles for bacteria-infected wounds. The SEM images revealed nanofibers with uniform size and oriented in 3D angle with the fiber diameter that ranged between 128 and 291 nm. The in vitro antimicrobial experiments showed that the nanofibers were very effective against bacterial strains with inhibition zones of 1.1 ± 0.05 and 1.3 ± 0.08 cm against *E. coli* and *B. circulans*, respectively. These results indicated that Ag nanoparticle-incorporated nanofibers demonstrated excellent antimicrobial activity, resulting from their large surface area, spherical shape, and small size [159].

Lee et al. formulated electrospun lignin–PVA nanofibers for wound healing applications. The FTIR and XRD analysis confirmed the successful fabrication of nanofibers. The SEM images of lignin–PVA nanofibers displayed an average diameter of 230 nm and a very tiny protrusion on the nanofiber surface. The mechanical characterization of lignin–PVA nanofibers showed breaking stress of 2.52 MPa and modulus of 8.9 MPa. The water uptake studies demonstrated water uptake and water retention of 359% and 208%, respectively, indicating a good ability to absorb and store wound exudates in the nanofiber wound dressings. The antimicrobial experiments of the nanofibers utilizing a standard test method demonstrated a significantly greater inhibitory effect against *S. aureus* bacterial strain than electrospun plain PVA nanofibers, which were used as a control [160].

### 5.10. Other Biopolymers Combined with PVA/PCL for Hybrid Nanofiber Fabrication

There are other biopolymers with very interesting properties that can be used for the fabrication of hybrid nanofibers, such as elastin, pectin, dextran, natural rubber, etc. Balakrishnan and Thambusamy fabricated electrospun β-Cyclodextrin–PVA nanofibers decorated with Ag nanoparticles and riboflavin for wound dressing application [161]. The mechanical characterization of nanofibers exhibited the highest tensile strength of about 75 MPa with the elongation at a break that ranges between 8% and 20%. The in vitro biodegradation studies showed that all the nanofibers degradation increased as the incubation period increased, and the presence of Ag nanoparticles and riboflavin in the nanofibers influenced the degradation rate of the nanofibrous scaffolds. The in vitro cytotoxicity studies were performed using MTT assay showed more than 90% cell viability of HEK-293 cells for pure nanofibers and decreased to 75% for the drug-loaded nanofibers, indicating very little toxicity of dual-drug loaded nanofibers. The antimicrobial analysis of dual-drug loaded cyclodextrin–PVA nanofibers demonstrated excellent antagonistic bactericidal activity efficacy against both *S. aureus* and *E. coli,* while pristine nanofibers did not display any significant antibacterial efficacy. The wound healing assessment in vivo using wounds on albino mice revealed percentage wound closure of 56%, 63%, 75%, 87%, and 98%, for reference, pristine nanofibers, Ag nanoparticle nanofibers, riboflavin nanofibers, and dual-drug loaded nanofibers on the 10th day of surgery, respectively [161].

Azarian et al. developed chloroacetated natural rubber–PVA nanofibers loaded with kaolin and starch via green electrospinning technique for wound dressing application. The in vitro cytotoxicity studies of the nanofibers showed that after the incorporation of kaolin and starch in the nanofibers, the cell viability was 100% when incubated with human dermal fibroblast cells, indicating their excellent biocompatibility [162]. Yang et al. reported polymeric nanofibers prepared from Konjac glucomannan and PVA for skin regeneration. The mechanical properties of the nanofibers were a tensile strength of approximately 2.5 MPa, which is suitable for wound dressing application. The in vivo wound closure experiments employing Sprague Dawley rats demonstrated that the healing ratio of the untreated wounds, pure PVA nanofibers, and Konjac glucomannan–PVA nanofibers reached 86%, 92%, and 98%, respectively, indicating that the addition of Konjac glucomannan significantly accelerated the wound healing process [163].

Alipour et al. formulated pectin–PVA–PVP-mafenide acetate electrospun nanofibers incorporated with Ag nanoparticles for wound healing. The FTIR, XRD, and Energy dispersive X-ray analysis (EDS) were employed and confirmed the successful formulation of the hybrid nanofibers. The Young’s modulus of the Ag nanoparticle-loaded pectin–PVA–PVP-Mafenide acetate nanofibers increased from 53.4 ± 4.6 to 63.4 ± 3.3 when the Ag content increased from 0.2 to 0.5 wt%, and the same finding was visible for elongation at break and tensile strength [164]. The in vitro cytotoxicity of the nanofibers employing MTT assay on HSF-PI 18 fibroblast cells showed a high cell viability of approximately 92%, which was decreased by a high content of Ag nanoparticles. The in vitro antimicrobial analysis exhibited high inhibition zone against *E. coli* and *P. aeruginosa* than *S. aureus* for Ag nanoparticle-loaded nanofibers when compared to reference the antibiotic, ampicillin. The wound healing studies in vivo using wounds on the back of white rabbits showed that the wounds dressed with hybrid nanofibers loaded Ag nanoparticles promoted rapid skin regeneration compared to the plain nanofibers at the end of day 12 [164]. Table 1 below shows the summary of biopolymer-based hybrid nanofibrous scaffolds.

## 6. Conclusions

The electrospinning of biopolymers to fabricate nanofibers offers interesting biomedical applications suitable for wound dressing, tissue engineering, and drug delivery. Some of the unique features of nanofibers are high porosity, biodegradability, biocompatibility, non-cytotoxicity, and exceedingly high surface area to volume ratio. However, nanofibers prepared from biopolymers suffer from poor mechanical properties, insignificant antibacterial effects, making them inappropriate for wound dressing and skin regeneration. The combination of biopolymers and synthetic polymers for the fabrication of nanofibers seems to be the most useful strategy to overcome the poor mechanical performance of biopolymer-based nanofibers. The fabrication of hybrid nanofibers using biopolymers and PVA/PCL showed excellent mechanical features (such as tensile strength, elongation at break, and others) that resemble the human skin and suitable for wound management. These biopolymer–PVA/PCL hybrid nanofibers were loaded with selected bioactive agents such as metal-based nanoparticles, plant extracts, antibiotics, and growth factors for enhanced biological activities such as antibacterial, antioxidant, and anti-inflammatory efficacy. The results obtained from the series of in vitro and in vivo studies are very promising, and there is no doubt that some of these dressings will be employed in clinics in the near future. Furthermore, these biopolymer-based nanofibrous scaffolds are suitable for wound healing and tissue regeneration applications.

## Figures and Tables

**Table 1:** Summary of biopolymer-based hybrid nanofibrous scaffolds.

Polymers Used	Loaded Bioactive Agents	Study Outcomes	Reference
Chitosan and PVA	Ag nanoparticles	High swelling capacity and accelerated wound healing	[56]
Chitosan and PVA	-	Good mechanical performance and excellent biocompatibility with high antibacterial effects	[57]
Chitosan and PVA	Cefadroxil	Sustained drug release and good antibacterial activity	[58]
Chitosan and PVA	Tetracycline	Good antibacterial efficacy and fast wound recovery	[59]
*N*-carboxyethyl chitosan and PVA	-	Non-toxicity	[60]
Chitosan and PVA	-	The accelerated diabetic wound healing process	[61]
Carboxymethyl chitosan and PVA	Au nanoparticles	Non-toxicity and high antibacterial effects	[62]
Chitosan and PVA	Silk protein sericin	Excellent biocompatibility and accelerated wound healing process	[63]
Chitosan and PVA	Ag and Au nanoparticles	Superior antimicrobial activity	[64]
Chitosan and PVA	-	Accelerated wound healing mechanism	[65]
Chitosan and PVA	Arthrospira platensis	High cell viability and potential wound healing process	[66]
Chitosan and PVA	Graphene oxide	Good bactericidal activity	[67]
Chitosan and PVA	Cu metal-organic frameworks	Excellent cell adhesion and proliferation with a fast wound healing process	[68]
Carboxyethyl chitosan and PVA	Chamomile	Good antioxidant and antibacterial activity	[69]
Chitosan and PVA	ZnO nanoparticles	Good antibacterial and fast diabetic wound healing	[70]
Chitosan and PVA	Halloysite nanotubes	Good biocompatibility and cell attachment	[71]
Chitosan and PVA	Nepeta dschuparensis and honey	Faster burn wound healing process	[2]
Chitosan and PVA	Honey	Superior antibacterial efficacy	[73]
Chitosan and PVA	Ag nanoparticles	Superior synergistic antibacterial effects	[74]
Chitosan oligosaccharides and PVA	Ag nanoparticles	High antibacterial efficacy and fast wound closure	[75]
Chitosan oligosaccharide and PVA	Ag nanoparticles	Accelerated wound healing process	[76]
Chitosan and PVA	-	Good wound healing properties	[77]
Chitosan and PVA	Ag nitrate and titanium	Excellent antibacterial activity	[78]
*N*-Maleoyl-functional chitosan and PVA	Tetracycline hydrochloride	Good wound-healing effects and superior antibacterial effects	[79]
Chitosan and PVA	-	Good cell adhesion and proliferation	[80]
Chitosan and PVA	-	Potential wound healing management	[81]
Chitosan and PVA	-	Accelerated wound healing process	[82]
Chitosan and PVA	Ag ions	Excellent antibacterial activity	[83]
Chitosan and PVA	Graphene oxide and ciprofloxacin	Good antibacterial efficacy and excellent cytocompatibility	[84]
Chitosan and PVA	-	Rapid burn wound healing process [74]	[85]
Chitosan and PVA	*Bidens pilosa*	Good antimicrobial activity	[86]
Chitosan and PVA	-	Fast wound healing recovery	[87]
Chitosan-graft polyaniline and PCL	-	Good mechanical properties and accelerated wound closure	[88]
Chitosan and PLA	Curcumin	Initial burst drug release followed by sustained release	[89]
Chitosan, PCL, and PVA	-	The rapid wound healing process	[90]
Chitosan and PCL	*Aloe vera*	Moderate WVTR and Excellent antibacterial activity	[91]
Chitosan and PCL	Nitric acid	Fast wound healing mechanism	[92]
Chitosan-g-polyaniline and PCL	-	Excellent antibacterial activity and good wound closure	[93]
Chitosan, PCL, and HA	-	Good biocompatibility and non-toxicity,	[94]
Chitosan and PCL	Resveratrol and ferulic acid	Faster wound contraction rate	[95]
Chitosan and PCL	-	Non-toxicity	[96]
Chitosan and PCL	*Aloe vera*	Good mechanical and biological properties	
Gelatin and PVA	ZM essential oil	Good cytocompatibility and antibacterial effects	[99]
Gelatin and PVA	*Carica papaya*	good biocompatibility and bactericidal activity	[100]
Gelatin, PVA, and chitosan	Glucantime	Good properties for the treatment of Leishmania wounds	[101]
Gelatin and PCL	Quercetin and ciprofloxacin	Initial burst drug release followed by sustained frug release with fast wound closure	[102]
Gelatin, PCL, and chitosan	Curcumin	High cell attachment and biocompatibility with good antioxidant efficacy	[103]
Gelatin and PCL	QAS	Excellent mechanical properties and high antibacterial activity	[104]
Gelatin and PCL	Amoxicillin and Zn nanoparticles	Sustained drug release profile, good antibacterial efficacy, and accelerated wound healing	[105]
Gelatin and PCL	Ketoprofen	High cell viability indicating good biocompatibility	[106]
Gelatin and PCL	Cerium oxide	Moderate WVTR and fast wound recovery	[107]
Gelatin and PCL	Halloysite nanotubes	Non-toxicity	[108]
Gelatin and PCL	Taurine	Accelerated wound healing process	[109]
Gelatin and PCL	Clove essential oil	Superior antibacterial activity	[110]
Gelatin and PCL	*Gymnema sylvestre*	Initial burst release that can contribute to good antibacterial effects	[111]
Gelatin and PCL	Human urine-derived stem cells	Improved wound healing properties and increased re-epithelization	[112]
Gelatin and PCL	Lawsone	Good mechanical properties, superior antibacterial efficacy, and accelerated wound healing process	[113]
Sodium Alginate and PVA	Dexpanthenol	Controlled drug release and good cytocompatibility	[115]
Alginate, PVA, and Chitosan	Asiaticoside	Improved wound healing mechanism	[116]
Sodium Alginate and PVA	ZnO nanoparticles	Excellent antibacterial activity	[117]
Alginate and PVA	Gatifloxacin	Continuous controlled drug release mechanism	[118]
Sodium Alginate and PVA	Moxifloxacin	High swelling capacity, good antibacterial efficacy, and superior wound healing process	[119]
Alginate and PCL	Nanocrystal cellulose	Non-toxicity	[120]
Alginate and PVA	-	Surface morphology that mimics ECM	[121]
Alginate and PVA	-	Superior wound healing mechanism	[122]
Sodium Alginate and PVA	-	Accelerated wound healing process	[123]
Cellulose and PVA	Curcumin	Excellent biocompatibility and fast wound healing process	[126]
Hydroxyethyl cellulose and PVA	-	Good mechanical properties and non-toxicity	[127]
Cellulose acetate and PCL	Metallic nanoparticles (Ag, CuO, and ZnO nanoparticles)	Good antibacterial activity	[128]
Cellulose acetate and PCL	Propolis	Excellent antioxidant and antimicrobial efficacy	[129]
HA and PVA	-	Good cytocompatibility and fast wound healing	[132]
Hyaluronate-methacrylated and PVA	-	Non-toxicity and high cell adhesion	[133]
Hyaluronan and PCL	Epidermal growth factors	Accelerated wound healing	[134]
Collagen and PVA	Graphene oxide	Excellent biocompatibility and improved wound healing process	[138]
Collagen and PVA	-	High swelling capacity and good cytocompatibility	[139]
Collagen and PCL	-	Higher cell proliferation and migration rate	[140]
Collagen and PCL	*N*-acetylcysteine	Initial rapid drug release followed by sustained release, and a fast wound healing process	[141]
Collagen and PCL	Doxycycline	Good biocompatibility	[142]
Gum tragacanth and PVA	-	High cell adhesion and proliferation, and good antibacterial efficacy	[144]
Gum tragacanth and PVA	Curcumin	High cell attachment and proliferation	[145]
Gum Arabic and PVA	Ag nanoparticles	Non-toxicity and excellent antimicrobial activity	[146]
Gum tragacanth–PCL–PVA	-	Good mechanical performance and accelerated diabetic wound closure	[147]
Gum tragacanth and PCL	Curcumin	Initial burst drug release followed by a sustained release	[148]
Gum tragacanth and PCL	*Aloe vera*	High cell proliferation	[149]
Gum tragacanth and PCL	Curcumin	Accelerated diabetic wound healing process	[150]
Silk fibroin and PVA	Epidermal cells	Fast wound recovery	[153]
Silk fibroin and PVA	Starch nanoparticles and *Aloe vera*	High encapsulation efficiency and good antioxidant efficacy	[154]
Silk fibroin and PVA	Amoxicillin trihydrate	Improved mechanical properties and excellent antibacterial activity	[155]
Silk fibroin and PCL	-	High antibacterial effects	[156]
Silk fibroin and PVA	-	Accelerated diabetic wound healing process	[157]
Lignin and PVA	Ag nanoparticles	Good antimicrobial efficacy	[159]
Lignin and PVA	-	Enhanced mechanical properties and good antibacterial activity	[160]
β-Cyclodextrin and PVA	Ag nanoparticles and riboflavin	Non-toxicity, excellent antagonistic bactericidal activity, and fast wound healing process	[161]
Chloroacetated natural rubber and PVA	Kaolin and starch	Excellent cytocompatibility	[162]
Konjac glucomannan and PVA	-	Accelerated wound healing mechanism	[163]
Pectin, PVA, and PVP	Ag nanoparticles	Good cytocompatibility, higher antibacterial efficacy, and accelerated wound healing process	[164]

## Data Availability

All supporting data are reported in the manuscript.
